# Identification of shared molecular biomarkers and pathogenic mechanisms between gastroesophageal reflux disease and ischemic stroke via integrated machine learning

**DOI:** 10.1097/MD.0000000000046014

**Published:** 2025-11-21

**Authors:** Fang Huang, Jie Zhang

**Affiliations:** a Community Health Service Center of Cangqian Sub-district, Yuhang District, Hangzhou City, Zhejiang, China; b Department of Rehabilitation Medicine, Tongde Hospital of Zhejiang Province, Hangzhou, Zhejiang, China.

**Keywords:** biomarker, gastroesophageal reflux disease, ischemic stroke, LASSO, machine learning

## Abstract

Growing epidemiological evidence suggests a bidirectional relationship between gastroesophageal reflux disease (GERD) and ischemic stroke (IS), yet the shared molecular mechanisms remain poorly characterized. This study aims to identify common biomarkers and elucidate the pathogenic links between GERD and IS using integrative bioinformatics and machine learning approaches. Transcriptomic datasets for GERD (GSE26886 and GSE39491) and IS (GSE22255 and GSE58294) were obtained from the Gene Expression Omnibus. Batch effects were corrected using ComBat, and shared differentially expressed genes were identified via limma. Functional enrichment analyses (gene ontology and Kyoto encyclopedia of genes and genomes) were performed to uncover involved pathways. Key hub genes were selected using 3 machine learning algorithms: least absolute shrinkage and selection operator, support vector machine with recursive feature elimination, and random forest. Diagnostic utility was assessed through receiver operating characteristic curve analysis. We identified 52 upregulated and 57 downregulated differentially expressed genes common to both diseases. Enriched pathways included IL-17 signaling, glycosphingolipid biosynthesis, and PI3K-Akt signaling. Machine learning integration revealed 9 hub genes (FAM46C, FUT4, ODC1, UQCRB, ID2, TSC22D1, IL17RB, AHR, and MGAT4B) with consistent dysregulation in GERD and IS. These genes demonstrated high diagnostic accuracy, with combined area under the curve values between 0.9 and 1.0 across validation cohorts. IL17RB and FUT4 were notably upregulated, suggesting roles in inflammatory and glycosylation pathways. Our findings reveal convergent molecular pathways and potential diagnostic biomarkers linking GERD and IS. The identified hub genes may serve as dual-purpose therapeutic targets aimed at mitigating shared inflammatory and vascular mechanisms. Further experimental validation is needed to confirm their clinical relevance.

## 1. Introduction

Gastroesophageal reflux disease (GERD) and stroke represent 2 prevalent yet pathophysiologically distinct conditions, with emerging evidence suggesting potential shared biological pathways. Observational studies have indicated that GERD may increase stroke risk (odds ratio: 1.22 for all-stroke, 1.19 for ischemic stroke),^[1,2]^ while bidirectional Mendelian randomization analyses reveal a causal interplay, with stroke subtypes (e.g., large-artery stroke, odds ratio: 1.49) also exacerbating GERD susceptibility.^[1,2]^ Despite these associations, the specific biomarkers and mechanistic links underlying this relationship remain poorly understood.

The pathogenesis of GERD involves multifactorial processes, including lower esophageal sphincter dysfunction, prolonged acid exposure, and inflammatory responses mediated by granulocytes or T-lymphocytes.^[3–5]^ Notably, these mechanisms may intersect with stroke pathways through shared risk factors such as hypertension (mediated effect reported in GERD-stroke association),^[1]^ obesity,^[6]^ and systemic inflammation.^[7]^ For instance, GERD-related esophageal mucosal damage triggers endoplasmic reticulum (ER) stress, which is implicated in both local inflammation and systemic vascular endothelial dysfunction.^[7]^ Additionally, humoral markers of asymptomatic lung injury in GERD patients^[8]^ suggest potential circulating biomarkers that could also reflect cerebrovascular injury.

Current research gaps include: limited identification of molecular biomarkers (e.g., calcitonin gene-related peptide upregulation in reflux hypersensitivity^[9]^) that may concurrently predict stroke risk; unclear mediation effects of cardiovascular risk factors (e.g., major depressive disorder shown to mediate GERD-stroke links^[10]^); and heterogeneity in GERD phenotypes (erosive vs nonerosive) and their differential associations with stroke subtypes.^[1,11]^ Machine learning approaches offer a promising solution to integrate multi-omics data (genetic, proteomic, and clinical) from existing genome-wide association studies^[2,12]^ and mechanistic studies,^[5,7]^ enabling the identification of high-dimensional patterns that traditional statistical methods may overlook.

**Figure 1.**
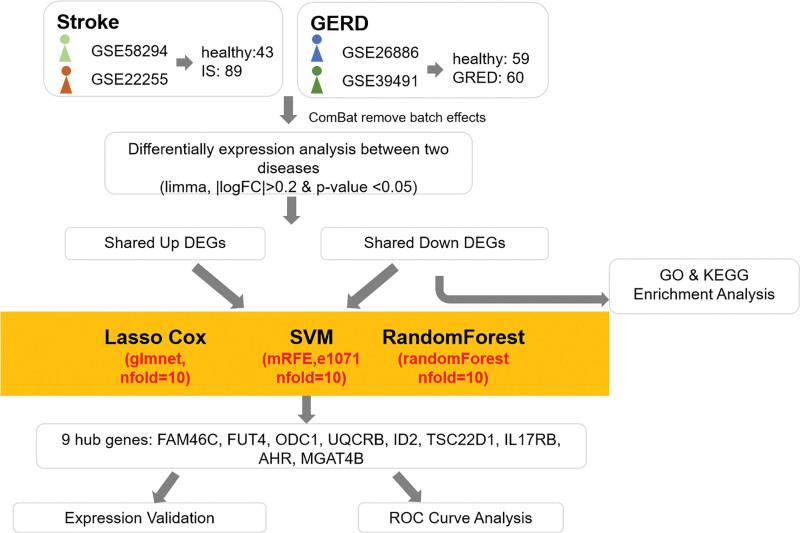
Flowchart of shared gene and pathway identification between ischemic stroke (IS) and gastroesophageal reflux disease (GERD).

This study systematically integrated gene expression datasets from the Gene Expression Omnibus (GEO) database for GERD and ischemic stroke. Common differentially expressed genes (DEGs) were identified through comprehensive differential expression analysis. To prioritize clinically relevant biomarkers, we employed least absolute shrinkage and selection operator (LASSO) regression, support vector machine-recursive feature elimination, and random forest algorithms in a complementary analytical framework. This multi-method approach not only circumvents the limitations inherent to single-algorithm strategies but also enhances result reliability through rigorous cross-validation procedures. Figure 1 illustrates a schematic representation of the integrated workflow.

## 2. Materials and methods

### 2.1. Data download and preprocessing

Transcriptome profiling datasets were retrieved from the GEO database (https://www.ncbi.nlm.nih.gov/geo/) using the R package GEOquery (v2.76.0; Bioconductor Core Team, Seattle). The study incorporated 4 independent cohorts: GSE58294 and GSE22255 for ischemic stroke (IS) analysis, and GSE26886 and GSE39491 for GERD investigation. Detailed clinical characteristics and sample information of the datasets are summarized in Table 1. For each dataset, the corresponding platform files were also retrieved to obtain the probe-to-gene mapping information. The raw CEL files were extracted, and background correction, normalization, and summarization were performed using the rma function from the affy package (Bioconductor Core Team, Seattle). All datasets were derived from microarray platforms and were uniformly processed using the same normalization pipeline to ensure comparability across datasets. Probe intensities were summarized to gene level by taking the median value of multiple probes corresponding to the same gene. Probes mapping to multiple genes were excluded from the analysis. After data cleaning, low-abundance genes were filtered out based on expression levels across samples. The final expression matrices were log2-transformed and normalized between arrays using the normalizeBetweenArrays function from the limma package (Walter and Eliza Hall Institute of Medical Research [WEHI], Melbourne, Victoria, Australia). To address batch effects between datasets, the ComBat function from the sva package was applied. For IS datasets (GSE58294 and GSE22255), and GERD datasets (GSE26886 and GSE39491), the expression matrices were merged, and batch correction was performed based on the dataset origin. Principal component analysis was conducted before and after batch correction to assess the effectiveness of the ComBat method.

**Table 1:** Characteristics of the included ischemic stroke (IS) and gastroesophageal reflux disease (GERD) cohorts.

GEO accession	Platforms	Samples	Tissue
GSE58294	GPL570	69 cardioembolic stroke samples and 23 controls	Blood
GSE22255	GPL570	20 IS patients and 20 controls	Blood
GSE26886	GPL570	20 GRED and 19 controls	Barrett esophagus/esophageal squamous epithelium
GSE39491	GPL571	40 GRED and 40 controls	Barrett esophagus/normal mucosa from squamous esophagus

GEO = Gene Expression Omnibus, IS = ischemic stroke.

### 2.2. Differential expression analysis

Differential expression analysis was performed using the limma package. For each dataset, a linear model was fitted to the normalized expression data, and empirical Bayes moderation of the standard errors was applied. Contrasts were defined to compare case and control groups. The topTable function was used to extract DEGs with a *P*-value < .05 and |log2(fold change)| > 0.2. Volcano plots were generated to visualize significant DEGs, and heatmaps were created for the top DEGs using the pheatmap package.

### 2.3. Enrichment analysis

Gene ontology and Kyoto encyclopedia of genes and genomes (KEGG) pathway enrichment analyses were performed using the clusterProfiler package (Southern Medical University, Guangzhou, Guangdong, China). DEGs common to both IS and GERD were converted from gene symbols to Entrez IDs using the bitr function. For gene ontology enrichment, the entire human genome was used as the background. For KEGG enrichment, pathways with adjusted *P*-values < .05 were considered significant. The results were visualized using dot plots and bubble plots to highlight the most significantly enriched terms and pathways.

### 2.4. Machine learning for hub gene identification

Machine learning approaches were employed to identify hub genes from the DEGs. LASSO regression was implemented using the glmnet package with 10-fold cross-validation to determine the optimal regularization parameter (lambda.min), selecting features with nonzero coefficients at this threshold. Support vector machine with recursive feature elimination was implemented using the mRFE package with the e1071 backend, utilizing 10-fold cross-validation to identify the feature subset corresponding to minimum classification error. Random forest analysis was conducted using the randomForest package (Fortran Original by Leo Breiman & Adele Cutler; R port by Andy Liaw & Matthew Wiener) with 10 repetitions of 10-fold cross-validation to determine the optimal feature number based on minimal cross-validation error, assessing variable importance through mean decrease in accuracy. The final hub genes were defined as the intersection of genes selected by all 3 algorithms, ensuring robust and consensus feature selection across complementary machine learning paradigms.

### 2.5. Validation and receiver operating characteristic (ROC) analysis

The expression patterns of the identified hub genes were validated across all datasets. Boxplots were generated to compare gene expression levels between case and control groups, with statistical significance assessed using Wilcoxon rank-sum tests. The Wilcoxon rank-sum test was employed as the primary method for hypothesis testing because the normality assumption for parametric tests could not be guaranteed for all gene expression distributions across datasets. Subsequently, we evaluated the diagnostic performance of each individual hub gene and their combined predictive power using ROC curve analysis. For individual genes, ROC curves were constructed and the area under the curve (AUC) was calculated to quantify their discriminatory capacity. To harness the collective predictive power of all 9 hub genes, we developed a combined diagnostic model using multivariate logistic regression. The probability scores derived from this model were used to generate a composite ROC curve. The optimal cutoff threshold for the combined model was determined by maximizing the Youden index, balancing sensitivity and specificity.

## 3. Results

### 3.1. Identification of IS-associated genes

**Figure 2.**
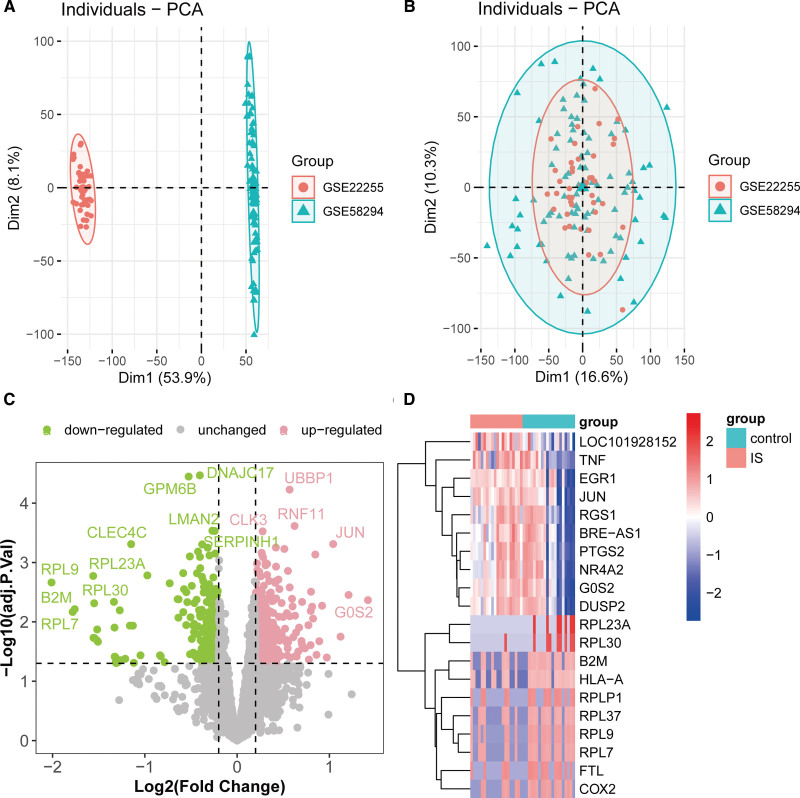
Identification of differentially expressed genes in IS. PCA results of the GSE22255 and GSE58294 cohorts before (A) and after (B) batch effect correction. (C) Volcano plot of differential expression analysis between IS and control groups in the GSE22255 cohort. (D) Top 20 upregulated and downregulated genes with the largest expression fold-changes in IS. IS = ischemic stroke.

Batch effects between the 2 IS cohorts (GSE22255 and GSE58294) were systematically corrected using ComBat (Fig. 2A and B). To identify IS-specific molecular signatures, differential expression analysis was performed on the GSE22255 dataset, which included transcriptomic profiles of peripheral blood mononuclear cells from 20 IS patients and 20 age- and sex-matched healthy controls. Limma R package-based analysis revealed 343 significantly upregulated genes and 335 downregulated genes in IS patients (Table S1, Supplemental Digital Content, https://links.lww.com/MD/Q706), with pronounced dysregulation observed for key genes including JUN, TNF, COX2, and RPL7 (Fig. 2C and D).

### 3.2. Identification of DEGs in GERD

**Figure 3.**
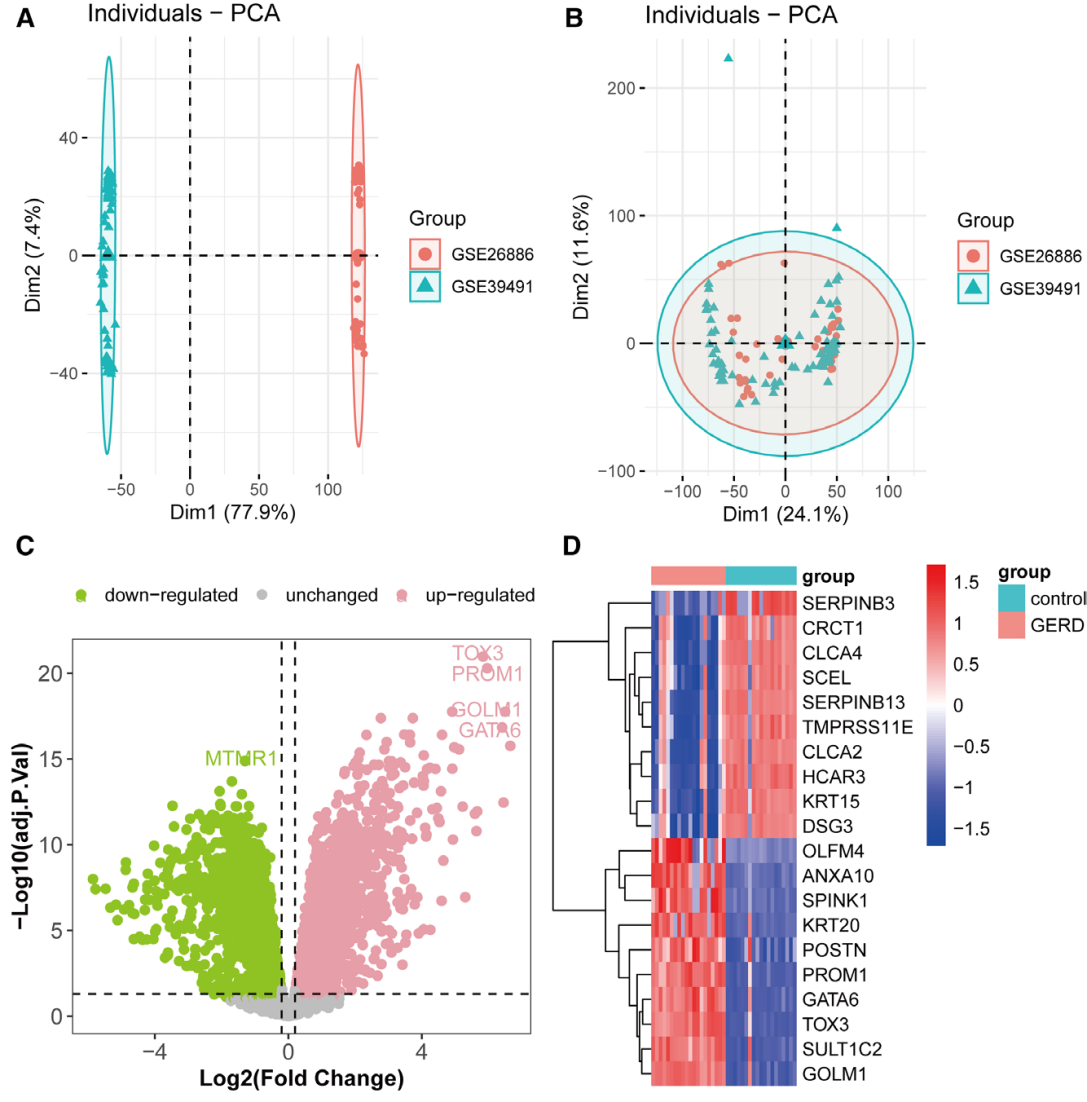
Identification of GERD-associated genes. PCA results of the GSE26886 and GSE39491 cohorts before (A) and after (B) batch effect correction. (C) Volcano plot of differential expression analysis between GERD and control groups in the GSE26886 cohort. (D) Top 20 upregulated and downregulated genes with the largest expression fold-changes in GERD. GERD = gastroesophageal reflux disease.

To further identify genes associated with GERD, we first performed batch effect correction on 2 GERD cohorts (GSE26886 and GSE39491) (Fig. 3A and B). Subsequently, differential expression analysis was conducted on the GSE26886 cohort, which included 20 GERD samples and 19 control samples. The results revealed that 2537 genes were significantly upregulated and 2796 genes were significantly downregulated in GERD patients compared with the control group (Fig. 3C, Table S2, Supplemental Digital Content, https://links.lww.com/MD/Q706). Among these, genes such as GOLM1, SULT1C2, and TOX2 exhibited the most pronounced upregulation, whereas SRPINB3, CRCT1, and CLCA4 displayed the most significant downregulation (Fig. 3D).

### 3.3. Shared DEGs and pathways between GERD and IS

**Figure 4.**
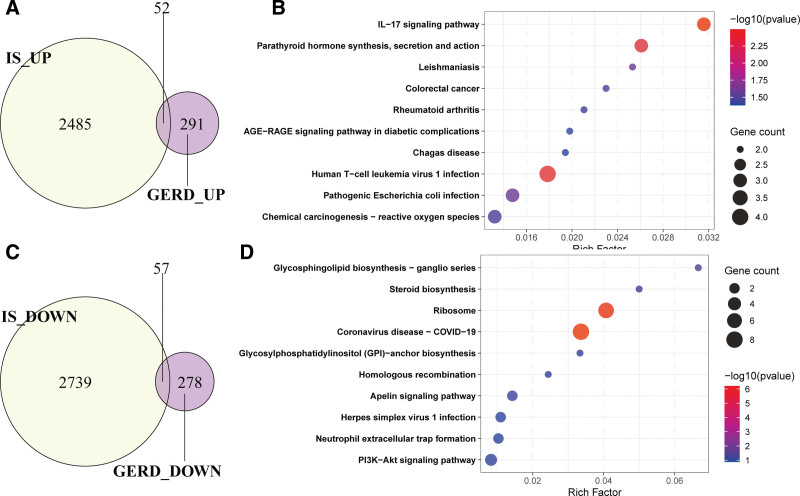
Shared upregulated/downregulated genes and pathways between IS and GERD. (A) Venn diagram showing 52 overlapping upregulated genes and (B) KEGG pathway enrichment results. (C) Venn diagram showing 57 overlapping downregulated genes and (D) KEGG pathway enrichment results. GERD = gastroesophageal reflux disease. IS = ischemic stroke, KEGG = Kyoto encyclopedia of genes and genomes.

The Venn diagram revealed 52 upregulated genes shared between IS and GERD (Fig. 4A). KEGG pathway enrichment analysis demonstrated that these overlapping genes were predominantly enriched in pathways such as the IL-17 signaling pathway, cancer-associated pathways, viral and bacterial infection-related pathways, and parathyroid hormone synthesis, secretion, and action (Fig. 4B). Additionally, 57 downregulated genes co-associated with both conditions were identified (Fig. 4C), which were significantly enriched in pathways including glycosphingolipid biosynthesis, steroid biosynthesis, ribosomes, glycosylphosphatidylinositol-anchor biosynthesis, homologous recombination, apelin signaling pathway, neutrophil extracellular trap (NET) formation, and PI3K-Akt signaling pathway (Fig. 4D).

### 3.4. Identification of 9 hub genes via machine learning

**Figure 5.**
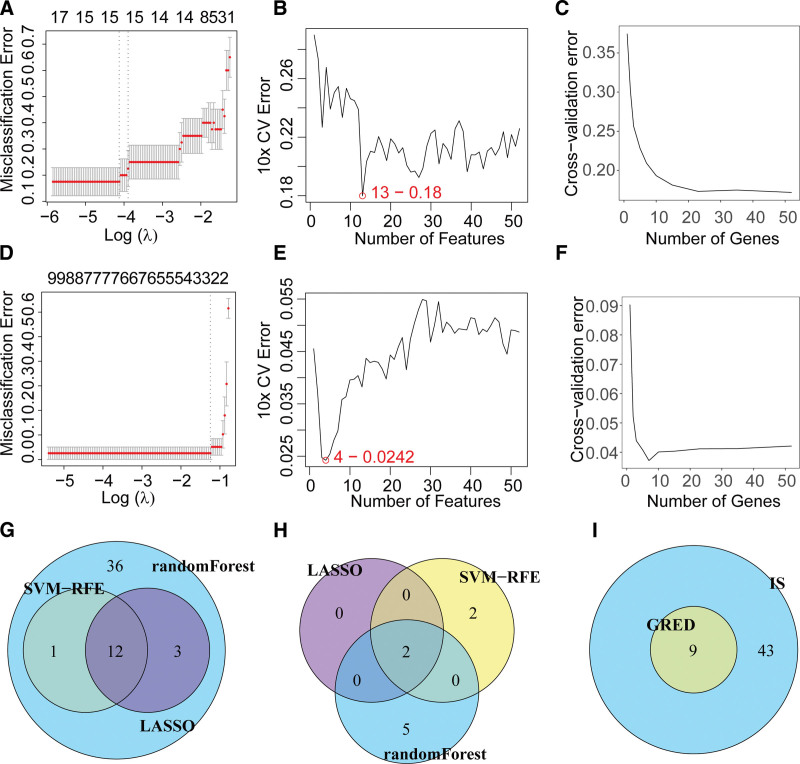
Identification of hub genes in ischemic stroke (IS) and gastroesophageal reflux disease (GERD) using multiple machine learning algorithms. (A–C) Feature selection and model optimization for identifying hub genes in IS using 3 machine learning approaches: (A) LASSO regression with cross-validation; the optimal λ value was selected based on minimum misclassification error. (B) Support Vector Machine with Recursive Feature Elimination (SVM-RFE); the number of features corresponding to the lowest 10 × cross-validation error (13 features, error = 0.18) was selected. (C) Random forest algorithm; the cross-validation error stabilizes after selecting approximately 20 genes, indicating convergence. (D–F) Feature selection and model optimization for identifying hub genes in GERD: (D) LASSO regression; the optimal λ value was determined by minimal misclassification error. (E) SVM-RFE; the optimal feature set was identified at 4 features (cross-validation error = 0.0242). (F) Random forest; cross-validation error plateaus after ~10 genes, suggesting sufficient feature selection. (G) Venn diagram of hub genes across IS algorithms. (H) Venn diagram of hub genes across GERD algorithms. (I) Venn diagram of 9 shared hub genes between IS and GERD. LASSO = least absolute shrinkage and selection operator, SVM-RFE = support vector machine with recursive feature elimination.

To further identify hub genes from these shared DEGs, we applied 3 machine learning algorithms. For IS, the LASSO, support vector machine, and random forest algorithms yielded 15, 13, and 52 hub genes, respectively (Fig. 5A–C). In GERD, the same algorithms identified 2, 4, and 7 hub genes, respectively (Fig. 5D–F). Taking the union of hub genes across algorithms, we obtained 52 hub genes for IS (Fig. 5G) and 9 hub genes for GERD (Fig. 5H). Finally, the intersection of these gene sets across both diseases revealed 9 shared hub genes: FAM46C, FUT4, ODC1, UQCRB, ID2, TSC22D1, IL17RB, AHR, and MGAT4B (Fig. 5I).

### 3.5. Validation of expression levels of the 9 hub genes

**Figure 6.**
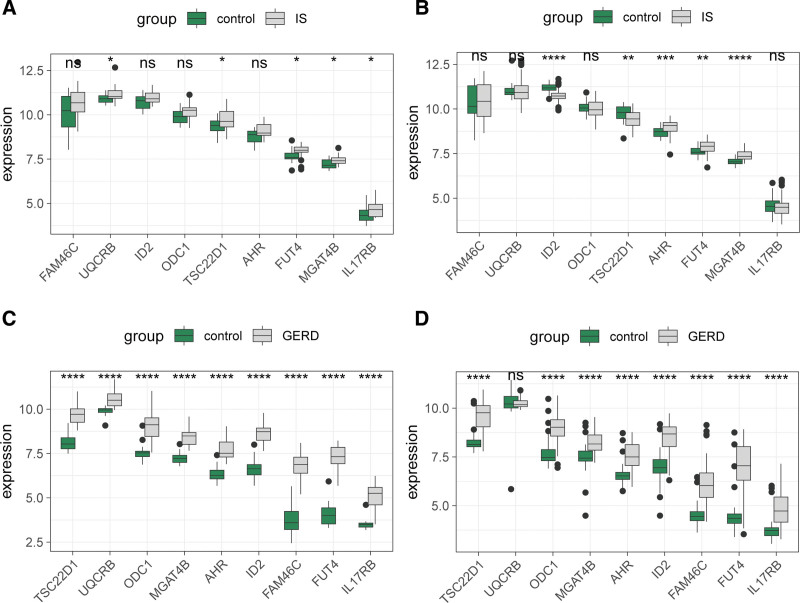
Expression validation of 9 hub genes. (A–B) Expression differences of hub genes between IS patients and controls. (A) GSE22255 dataset; (B) GSE58294 dataset. (C–D) Expression differences of hub genes between GERD patients and controls. (C) GSE26886 dataset; (D) GSE39491 dataset. Group comparisons were performed using Wilcoxon rank-sum tests. ns, not significant; * *P* < .05; ** *P* < .01; *** *P* < .001; **** *P* < .0001. GERD = gastroesophageal reflux disease, IS = ischemic stroke.

To further validate the expression patterns of the 9 shared hub genes in IS and GERD, we compared their differential expression between disease groups and control groups across all cohorts. The results revealed distinct expression profiles across different datasets: In the GSE22255 cohort, the expression levels of UQCRB, TSC22D1, FUT4, IL17RB, and MGAT4B were significantly upregulated in IS patients compared with controls (Fig. 6A). In the GSE58294 cohort, AHR, FUT4, and MGAT4B showed significant upregulation in IS, whereas ID2 and TSC22D1 were significantly downregulated relative to controls (Fig. 6B). In the GSE26886 cohort, all 9 hub genes exhibited significantly higher expression in GERD patients than in controls (Fig. 6C). In the GSE39491 cohort, all hub genes except UQCRB displayed significant upregulation in GERD patients compared with controls (Fig. 6D).

### 3.6. Diagnostic performance of hub genes in IS and GERD

**Figure 7.**
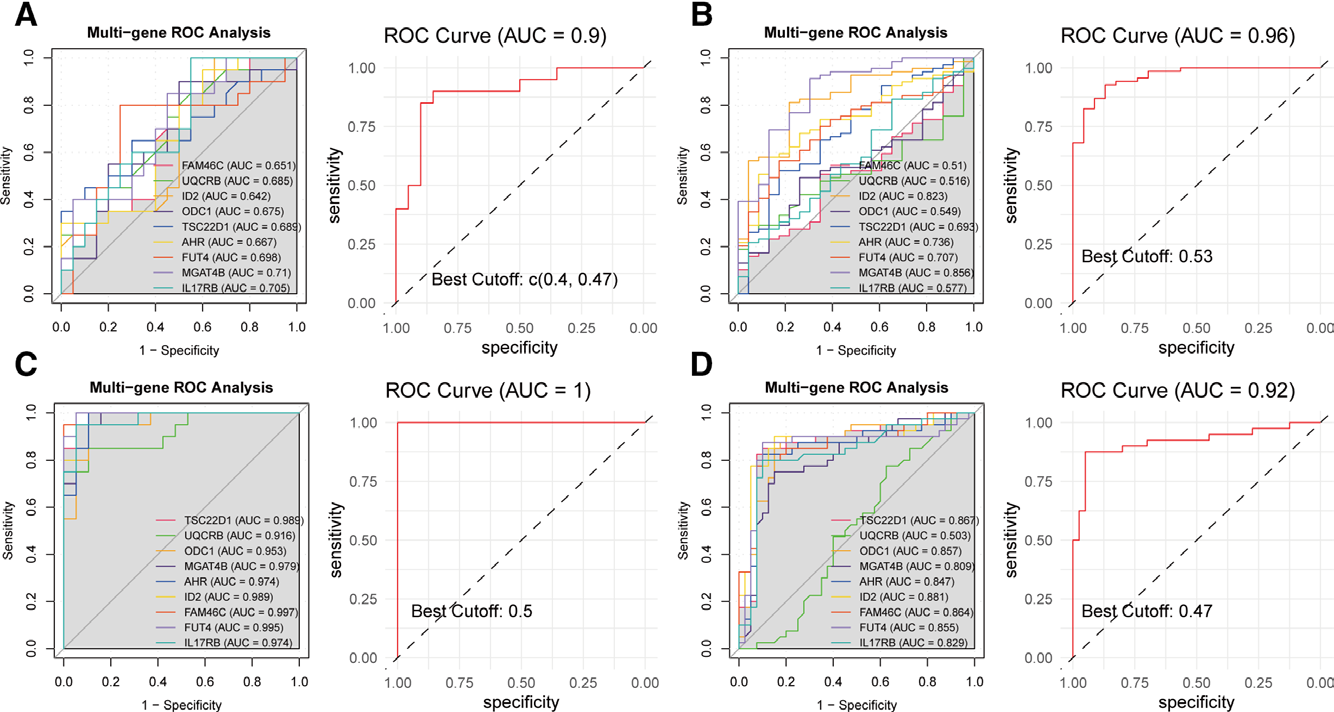
Diagnostic performance evaluation of the 9 hub genes in ischemic stroke and GERD cohorts. Receiver operating characteristic (ROC) curves demonstrating the discriminatory power of individual hub genes and their combined model for disease diagnosis. (A) ROC analysis in the GSE22255 ischemic stroke cohort. (B) ROC analysis in the GSE58294 ischemic stroke cohort. (C) ROC analysis in the GSE26886 GERD cohort. (D) ROC analysis in the GSE39491 GERD cohort. In each panel, ROC curves for individual genes are displayed in distinct colors in left, with the combined multivariate model represented by a red line in right. The dashed diagonal line indicates the reference line of random classification (AUC = 0.5). Corresponding AUC values for each gene and the combined model are provided in the legend. AUC = area under the curve, GERD = gastroesophageal reflux disease, ROC = receiver operating characteristic.

Finally, we evaluated the diagnostic performance of the 9 hub genes in IS and GERD. In the GSE22255 cohort, all genes exhibited AUC values > 0.6 for IS diagnosis, with the combined model achieving a diagnostic accuracy of 0.9 (Fig. 7A). In the GSE58294 cohort, ID2 (AUC = 0.823) and MGAT4B (AUC = 0.856) demonstrated strong individual performance, while the combined model showed superior diagnostic capacity (AUC = 0.96) (Fig. 7B). For the GERD cohort (GSE26886), all genes displayed AUC values > 0.9, indicating excellent diagnostic performance, and the combined model reached an AUC of 1 (Fig. 7C). In another GERD cohort (GSE39491), except for UQCRB (AUC = 0.503), all other genes exhibited AUC values > 0.8, showing favorable performance, and the combined model further improved to an AUC of 0.92 (Fig. 7D).

## 4. Discussion

GERD and IS impose significant global health burdens due to their high prevalence and complex pathogenesis. Epidemiological evidence suggests a potential bidirectional association between these disorders, yet molecular mechanisms underlying their co-occurrence remain poorly understood. This study identifies 9 hub genes (FAM46C, FUT4, ODC1, UQCRB, ID2, TSC22D1, IL17RB, AHR, and MGAT4B) as shared molecular signatures between GERD and stroke through integrative bioinformatics analysis. These genes are implicated in critical pathways such as IL-17 signaling, glycosphingolipid biosynthesis, and PI3K-Akt signaling, suggesting potential convergence of inflammatory and immune dysregulation in both diseases.

Previous studies have independently reported dysregulated pathways in GERD and stroke. For instance, oxidative stress-related genes (e.g., HO-1, GSH) are consistently implicated in GERD pathogenesis,^[13,14]^ while stroke studies highlight neuroinflammatory pathways (e.g., TNF-α, IL-6).^[15]^ However, our study is the first to systematically identify overlapping molecular mechanisms using machine learning-based integration of multi-omics data. Our study identified the IL-17 signaling pathway as a potential common pathogenic regulatory hub in both GERD and IS. Despite differences in their specific modes of action, IL-17-mediated processes in both diseases share a core proinflammatory response as a key characteristic. In GERD, IL-17 exacerbates clinical symptoms by activating acid-sensing receptors in esophageal squamous epithelial cells, thereby intensifying heartburn.^[16]^ Additionally, IL-17 upregulates the expression of proinflammatory genes (e.g., IL-17 receptors) to propagate the inflammatory cascade^[17]^ Notably, therapeutic interventions targeting the IL-17 pathway (such as STW5) alleviate inflammation by downregulating receptor expression, while omeprazole, though not directly affecting IL-17 levels, inhibits receptor activity.^[17]^ These findings underscore IL-17 signaling as a critical therapeutic target in GERD. In IS, IL-17 primarily drives neuroinflammation and blood–brain barrier (BBB) dysfunction. Studies demonstrate that IL-17A disrupts tight junction proteins (ZO-1, claudin-5, occludin), increasing BBB permeability. It further synergizes with proinflammatory cytokines (IL-6, TNF-α) to activate endothelial cell contraction and oxidative stress, promoting the infiltration of peripheral immune cells (neutrophils, Th17 cells) into the brain parenchyma and exacerbating neuronal damage.^[18]^ Moreover, IL-17-driven Th17/Treg imbalance establishes a positive feedback loop with microglial M1 polarization, sustaining inflammatory amplification. This process is closely linked to poststroke cognitive impairments (e.g., vascular dementia).^[18]^ Although IL-17-associated mechanisms exhibit tissue specificity (GERD primarily involves gastrointestinal inflammation, whereas IS focuses on cerebral microenvironmental dysregulation) their core pathology converges on IL-17-mediated chronic inflammation driven by Th17 cells. This shared feature suggests that IL-17 signaling may promote pathological damage across organs by enhancing systemic or local inflammatory microenvironments. For instance, IL-17-induced esophageal inflammation in GERD might indirectly disrupt cerebrovascular homeostasis via systemic inflammatory factors, while BBB breakdown in IS could facilitate inflammatory spread to the esophageal mucosal barrier. Furthermore, IL-17’s broad regulatory role in barrier function (e.g., intestinal and blood–brain barriers) may serve as a potential bridge for the comorbidity of these 2 diseases.

Previous investigations have highlighted that GERD significantly alters multiple metabolic pathways, including glycosphingolipid metabolism.^[19]^ Notably, another study reported that pathways associated with extracellular matrix–receptor interaction, xenobiotic metabolism, and glycosphingolipid metabolism were markedly activated as early as 3 hours poststroke,^[20]^ suggesting that glycosphingolipid metabolism may undergo dynamic changes during the acute phase of stroke. Dysregulation of glycosphingolipid metabolism has been linked to increased BBB permeability, inflammatory responses, and neuronal cell damage/repair processes. For instance, accumulation of specific glycosphingolipids can induce vascular wall inflammation, thereby promoting atherosclerosis (a key risk factor for stroke).^[21]^ Our study identified the PI3K-Akt signaling pathway as a critical regulator in both GERD and ischemic stroke. In a reflux esophagitis rat model, activation of the PI3K/Akt pathway was associated with oxidative stress and inflammatory injury; inhibition of this pathway alleviated inflammation and oxidative damage, thereby improving symptoms of reflux esophagitis.^[22]^ In the early stages of IS, PI3K/Akt activation promotes neuronal survival and regeneration, mitigates brain tissue injury, and exerts neuroprotective effects. Specifically, Akt activation suppresses the expression of apoptosis-related proteins, reduces apoptotic cell death, and alleviates cerebral ischemia–reperfusion injury. Additionally, this pathway regulates poststroke angiogenesis and neural plasticity, which are critical for brain tissue repair and functional recovery. However, aberrant PI3K/Akt activation may also drive pathological outcomes, such as glial cell proliferation and excessive inflammation, exacerbating stroke-induced tissue damage.^[23,24]^ Collectively, these findings suggest that precision-targeted strategies modulating the PI3K-Akt pathway may provide novel avenues for investigating the shared pathogenic mechanisms of GERD and stroke, as well as developing dual-purpose therapeutic interventions.

Our study revealed that NETs may play a critical role in both GERD and IS through shared inflammatory-thrombotic mechanisms. The excessive release of NETs could underlie the association between these 2 diseases: in GERD, chronic gastric acid reflux induces neutrophil activation and NETosis, with NET components (e.g., citrullinated histone H3, myeloperoxidase) directly damaging the esophageal mucosa and promoting local fibrosis.^[25,26]^ In IS, NETs exacerbate cerebral microthrombosis by activating platelets and promoting fibrin deposition, while simultaneously releasing proinflammatory cytokines (e.g., IL-6, TNF-α) that disrupt the BBB and amplify neurological injury.^[27]^ Notably, NETs may act as a bridge linking the 2 conditions: NET components in the esophageal microenvironment of GERD patients (e.g., circulating free DNA) could accelerate atherosclerosis via systemic inflammation,^[28]^ whereas poststroke neuroinflammation might worsen GERD symptoms through vagal nerve reflex, forming a vicious cycle.^[29]^ Therapeutic strategies targeting NETs (such as the PAD4 inhibitor Cl-amidine) have shown reduced mortality in animal models of ischemic stroke, highlighting the potential to explore multi-target interventions that simultaneously regulate NET-mediated inflammatory and thrombotic pathways.

Our integrated analysis positions ID2 as a pleiotropic regulator linking GERD and IS through shared pathways of cellular stress and immunomodulation. In IS, ID2 demonstrates a context-dependent role: while its expression is upregulated by hypoxia/ischemia, its silencing attenuates neuronal apoptosis and improves neurological outcomes, suggesting a involvement in stress-induced cell death pathways.^[30,31]^ Concurrently, ID2 is a critical determinant of T-cell fate, where its expression level dictates the balance between effector and exhausted CD8+ tissue-resident memory T cells during chronic CNS inflammation.^[32,33]^ In GERD, ID2 is essential for maintaining intestinal epithelial identity by repressing foregut transcription factors, and its deficiency leads to gastric metaplasia in the small intestine, a process akin to the mucosal changes in Barrett esophagus.^[34]^ This confluence of roles (regulating neuronal survival, T-cell exhaustion, and epithelial cell identity) suggests that ID2 dysregulation may simultaneously exacerbate cerebrovascular injury in IS and impair mucosal integrity in GERD, potentially creating a vicious cycle of inflammation and tissue damage via the brain–gut axis.

UQCRB, a subunit of mitochondrial complex III, may contribute to both diseases through dysregulated reactive oxygen species (ROS) accumulation and impaired energy metabolism. In GERD, UQCRB dysfunction could exacerbate esophageal mucosal injury by promoting oxidative stress. UQCRB is a key regulator of mitochondrial reactive oxygen species (mROS) production,^[35]^ and mROS-mediated oxidative stress is a central mechanism in GERD pathogenesis. In IS, UQCRB’s role is more complex and context-dependent. On one hand, UQCRB enhances angiogenesis through mROS-mediated HIF-1α signal transduction and VEGF expression,^[36,37]^ a process with dual effects post-ischemia (potentially beneficial for revascularization but detrimental if excessive, contributing to cerebral edema). UQCRB also positively regulates VEGFR2 signaling in endothelial cells,^[37]^ influencing cerebrovascular function. Notably, UQCRB is significantly downregulated in atherosclerosis,^[38]^ a major risk factor for IS. UQCRB upregulates COX5A, enhancing mitochondrial membrane potential, boosting ATP production, reducing ROS levels, decreasing secretion of inflammatory cytokines (TNF-α, IL-1β, IL-6), and reducing apoptosis rates,^[38]^ all mechanisms relevant to cerebrovascular protection. AHR, a ligand-activated transcription factor and a key regulator of inflammatory responses, may bridge GERD and IS via modulation of proinflammatory cytokines and immune cell function. In GERD, AHR may be involved in the response to environmental factors or endogenous ligands. AHR is highly expressed in gastric cancer tissues,^[39]^ and its signaling can be activated by various environmental pollutants, suggesting a potential role in esophageal inflammation and mucosal damage related to GERD. In IS, AHR’s mechanisms are more delineated. AHR plays a critical role in regulating neuroinflammation by influencing microglial polarization. AHR activation promotes a proinflammatory M1 phenotype, while its inhibition favors an anti-inflammatory M2 phenotype.^[40]^ Furthermore, AHR interacts with the TLR4 signaling pathway, modulating inflammation in cerebral ischemia/reperfusion injury.^[41]^ Notably, AHR inhibitors like Isorhapontigenin can alleviate CIRI by targeting this pathway.^[41]^ Drugs like Edaravone dexborneol ameliorate cognitive impairment by regulating the NF-κB pathway through AHR and promoting microglial M2 polarization.^[40]^ AHR also influences platelet activation and thrombosis,^[42]^ impacting IS pathology, and modulates ferroptosis in neuronal damage via the AHR-CYP1B1 axis.^[43]^

FUT4, a key enzyme synthesizing the CD15/sialyl Lewis X glycan epitope, emerges as a significant molecular bridge between GERD and IS, primarily through its roles in cell adhesion, inflammation, and barrier dysfunction. In GERD, FUT4-mediated fucosylation critically regulates the function of adhesion molecules, such as CD44, by modifying their glycostructures, which can alter cell–cell and cell–matrix interactions.^[44]^ The observed upregulation of FUT4 may lead to aberrant fucosylation of membrane proteins on esophageal epithelial cells, potentially disrupting the assembly and function of tight junctions, thereby impairing mucosal cohesion and repair, and rendering the epithelium more vulnerable to acid and pepsin injury. Furthermore, FUT4 expression is regulated by and can activate the Wnt/β-catenin signaling pathway,^[44,45]^ a pathway implicated in epithelial proliferation and inflammation. Dysregulated FUT4 might thus contribute to the aberrant inflammatory and proliferative responses characteristic of chronic GERD. In IS, FUT4’s role is multifaceted and predominantly pro-pathogenic. Firstly, its involvement in endothelial activation is critical. FUT4-mediated fucosylation of selectins and other adhesion molecules on endothelial cells and leukocytes is a well-established mechanism for promoting the firm adhesion and subsequent transmigration of inflammatory cells across the BBB. Secondly, beyond inflammation, FUT4 drives malignant phenotypes in various cells through the activation of the RAF-MEK-ERK and Wnt/β-catenin signaling pathways.^[46,47]^ In neurons and glial cells, such constitutive signaling activation could promote excitotoxicity, suppress pro-survival pathways, and ultimately exacerbate ischemic cell death.

As the rate-limiting enzyme in polyamine biosynthesis, ODC1 is upregulated in macrophages in response to inflammatory stimuli and functions to attenuate the production of proinflammatory cytokines and inhibit ROS-induced apoptosis, suggesting a protective feedback mechanism.^[48]^ Conversely, the loss of ODC1 promotes macrophage pyroptosis, a highly inflammatory form of cell death, exacerbating organ injury in septic models.^[49]^ In GERD, dysregulated ODC1 could disrupt the delicate balance of mucosal repair and inflammatory responses in the esophageal epithelium. In IS, altered ODC1 activity may similarly influence microglial activation and neuronal survival post-ischemia, positioning it as a key modulator of the shared inflammatory microenvironment in both conditions. TSC22D1 emerges as a pivotal transcriptional regulator with context-dependent roles in cell fate, potentially contributing to both GERD and IS through pathways involving cellular senescence and endothelial dysfunction. This gene encodes protein isoforms that can exert opposing effects on cell survival and proliferation, with one isoform inducing apoptosis and another suppressing it.^[50]^ Furthermore, TSC22D1 has been identified as a critical effector in oncogene-induced senescence, a key tumor-suppressor mechanism.^[51]^ More recently, TSC22D1 was shown to promote liver sinusoidal endothelial cell dysfunction and drive proinflammatory M1 macrophage polarization, thereby exacerbating tissue fibrosis.^[52]^ In GERD, TSC22D1 dysregulation could therefore influence esophageal epithelial cell turnover, apoptosis, and the development of fibrotic strictures. In IS, its role in promoting endothelial dysfunction and a proinflammatory macrophage phenotype aligns with mechanisms of blood-brain barrier disruption and chronic neuroinflammation, highlighting its involvement in fundamental stress–response pathways common to both diseases. IL17RB, a receptor for IL-17E (IL-25), is identified as a central node in type 2 inflammatory signaling, providing a direct mechanistic link between allergic/inflammatory pathways and the pathophysiology of GERD and IS. Its expression is strongly induced by the Th2 cytokine IL-4, creating a positive autocrine loop that sustains its own expression and amplifies inflammatory signaling, often through the NF-κB pathway.^[53]^ IL17RB is significantly upregulated during natural allergen exposure in conditions like seasonal allergic rhinitis, underscoring its role in environmental antigen-driven inflammation.^[54]^ In cancer contexts, IL17RB signaling promotes stemness and confers resistance to therapy.^[55]^ Within the GERD–IS axis, IL17RB likely mediates the activation of immune-esophageal epithelial interactions, exacerbating reflux-related inflammation. Simultaneously, in IS, its engagement could potentiate IL-25-driven neuroinflammation and impair brain repair mechanisms, positioning IL17RB as a key amplifier of pathogenic immune responses across organs.

FAM46C, identified as a noncanonical poly(A) polymerase that regulates mRNA stability and translation,^[56,57]^ may serve as a critical link between GERD and IS through its roles in maintaining cellular homeostasis. In GERD, the integrity of the esophageal mucosal barrier is paramount. FAM46C, by stabilizing mRNAs of specific target genes, could be essential for the continuous renewal and repair of the esophageal epithelium. Its downregulation, as suggested by our data, might lead to impaired stability of transcripts encoding for epithelial junction proteins or cytoprotective factors, thereby rendering the mucosa more susceptible to acid-peptic injury.^[58]^ Furthermore, FAM46C has been demonstrated to inhibit autophagy and promote the accumulation of protein aggregates, exacerbating ER stress.^[59,60]^ Given that ER stress is a known mechanism in GERD pathogenesis,^[7]^ loss of FAM46C function could amplify this stress response, leading to enhanced epithelial cell damage and impaired healing. This is supported by its established tumor-suppressor role in gastrointestinal cancers, where its loss promotes disease progression.^[58,61]^ In IS, the neuroprotective potential of FAM46C may be 2-fold. First, its role in inhibiting apoptosis is highly relevant. Studies have shown that FAM46C overexpression can suppress apoptosis induced by various stressors.^[57,62]^ In cerebral ischemia, the downregulation of FAM46C could therefore disrupt this protective function, permitting the widespread activation of apoptotic pathways in neuronal cells. Second, FAM46C’s function extends to regulating critical cellular processes like inflammation and organelle homeostasis. It has been identified as an interferon-stimulated gene that modulates inflammatory pathways,^[59]^ and its expression promotes mitochondrial and lysosomal components essential for cellular health.^[56]^ In IS, where neuroinflammation and mitochondrial dysfunction are central to secondary injury, a deficiency in FAM46C could exacerbate these damaging processes. Additionally, its interaction with and inhibition of Plk4 kinase,^[63,64]^ a regulator of centrosome duplication and the actin cytoskeleton, suggests a potential role in maintaining cytoskeletal integrity of cerebrovascular endothelial cells or neurons under ischemic stress. The convergent dysregulation of FAM46C in both GERD and IS suggests it may act as a pleiotropic regulator at the interface of epithelial integrity, neuronal survival, and inflammatory control. Its potential role in modulating the stability of mRNAs central to both diseases positions FAM46C as a compelling candidate for further mechanistic investigation into the gut–brain axis connecting GERD and stroke.

Several limitations should be acknowledged. First, the reliance on publicly available datasets introduces heterogeneity in sample collection (e.g., peripheral blood vs. tissue biopsies) and population demographics. Crucially, we were unable to perform sex-stratified analyses to explore potential gender-specific molecular mechanisms, which may be important given the known epidemiological differences in both GERD and IS between men and women. Furthermore, the lack of granular clinical subtyping represents another constraint. For ischemic stroke, we could not differentiate between etiological subtypes (e.g., large-artery atherosclerosis, cardioembolism, small-vessel occlusion), nor could we distinguish between erosive and nonerosive reflux disease phenotypes within the GERD cohorts. This limits our understanding of whether the identified biomarkers and pathways are universally applicable or specific to certain disease subtypes. Second, the cross-sectional design precludes causal inference between hub gene dysregulation and disease progression. Third, the exceptional diagnostic performance (AUC approaching 1.0) of our hub genes, particularly within the discovery cohorts, must be interpreted with caution. While this indicates strong separability between case and control groups in the analyzed datasets, it raises the possibility of model overfitting, especially given the current absence of a large, completely independent validation cohort. The high AUC values may be influenced by the relatively small sample sizes and the stringent feature selection process that optimized performance on the available data. Fourth, functional validation of candidate genes in cellular/animal models was beyond the scope of this study. Future directions include prospective cohort studies with larger sample sizes to validate the temporal dynamics and generalizability of hub gene expression, alongside intervention trials targeting IL-17/PI3K-Akt axes. Crucially, validating these biomarkers in an independent, external cohort will be essential to confirm their true diagnostic utility and mitigate concerns regarding overfitting. Multi-omics integration (e.g., proteomics, metabolomics) and single-cell sequencing could elucidate cell-type-specific contributions to shared pathophysiology.

## 5. Conclusion

In conclusion, this study elucidates shared molecular mechanisms underlying GERD and IS through integrative machine learning and systems biology approaches. The identified hub genes and pathways provide a foundation for developing novel diagnostic biomarkers and therapeutic targets. Further experimental validation and mechanistic dissection are warranted to translate these findings into clinical practice, ultimately improving outcomes for patients with cardioesophageal multimorbidity.

## Author contributions

**Conceptualization:** Fang Huang, Jie Zhang.

**Data curation:** Fang Huang, Jie Zhang.

**Formal analysis:** Fang Huang, Jie Zhang.

**Funding acquisition:** Fang Huang, Jie Zhang.

**Investigation:** Fang Huang, Jie Zhang.

**Methodology:** Fang Huang, Jie Zhang.

**Project administration:** Fang Huang, Jie Zhang.

**Resources:** Fang Huang, Jie Zhang.

**Software:** Fang Huang, Jie Zhang.

**Supervision:** Fang Huang, Jie Zhang.

**Validation:** Fang Huang, Jie Zhang.

**Visualization:** Fang Huang, Jie Zhang.

**Writing – original draft:** Fang Huang, Jie Zhang.

**Writing – review & editing:** Fang Huang, Jie Zhang.

## Supplementary Material


